# Unveiling the toxic effects of perfluorooctanoic acid on osteoblast function and extracellular matrix deposition using 2D and 3D models

**DOI:** 10.1038/s41420-025-02863-5

**Published:** 2026-01-09

**Authors:** Fiorenza Sella, Caterina Licini, Marta Lombó, Christian Giommi, Damiano Carbonari, Monica Mattioli-Belmonte, Oliana Carnevali

**Affiliations:** 1 Department of Life and Environmental Sciences, Università Politecnica delle Marche, Ancona, Italy; 2 INBB-Biostructures and Biosystems National Institute, Rome, Italy; 3 Department of Clinic and Molecular Science, Università Politecnica delle Marche, Ancona, Italy; 4 Department of Molecular Biology, Universidad de León, León, Spain; 5 INAL-Department of Technological innovations and Safety of Plants, Production and Anthropic Settlements, Rome, Italy; 6 INAIL-Marche Regional Directorate-Local Unit for Certification, Verification and Research of Ancona, Ancona, Italy; 7 Advanced Technology Center for Aging Research, IRCCS INRCA, Ancona, Italy

**Keywords:** Bone development, Differentiation

## Abstract

Emerging evidence revealed an association between perfluorooctanoic acid (PFOA) exposure and reduced bone mass density, leading to osteoporosis disease. This confirms the bone as a target tissue for per- and polyfluoroalkyl substances (PFAS). However, it is still unclear during which phase, proliferation or differentiation, PFOA exerts the most significant harm on osteoblasts, the cells responsible for secreting bone matrix. To tackle the intriguing question of how PFOA treatment affects the process, this study investigated the impact of different concentrations of PFOA on 2D and 3D human fetal osteoblast (hFOB1.19) cell line cultures representing the proliferation and differentiation phases, respectively. In 2D cultures, a 6-day PFOA exposure impaired antioxidant defense without directly altering osteogenesis or calcium deposition. In 3D spheroids, PFOA disrupted spheroid morphology and the deposition of the organic component of extracellular matrix (ECM) in a time-dependent manner. Given the relevance of the endocannabinoid system (ECS) in bone remodeling, we further assessed cannabinoid receptor 1 (CB1) levels. In 2D cultures, 10 µM PFOA reduced CB1 protein levels in parallel with decreased collagen levels. Conversely, in 3D spheroids, exposure to 100 µM PFOA for 2 days significantly increased CB1 levels while reducing the levels of degraded collagen. These findings emphasize the non-monotonic, phase- and time-dependent effects of PFOA on osteoblast function and ECM deposition, underscoring the need for further research into its long-term impact on bone homeostasis and human health induced by this emerging concern contaminant.

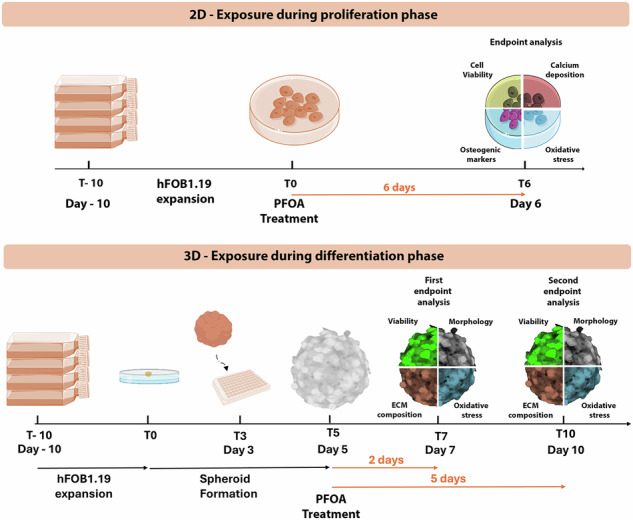

## Introduction

Bone is a dynamic and heterogeneous tissue consisting of osteoblasts (OBs), osteocytes, and osteoclasts (OCs), and bone extracellular matrix (ECM). Around 40% of ECM is made by organic components and 60% by an inorganic component. The organic components provide elasticity and flexibility and are made of 90% collagen type I (COL1), with the remaining 10% consisting of non-collagenous proteins, including osteocalcin (OCN), osteonectin, osteopontin, sialoprotein, cytokine, and growth factors such as transforming growth factor, insulin-like growth factor, fibroblast growth factor, and bone morphogenetic proteins. In contrast, the inorganic component, primarily composed of hydroxyapatite crystals, provides stiffness and strength to the bone [1–3]. OBs are crucial in secreting ECM during biomineralization, contributing to bone formation and homeostasis [4]. Bone homeostasis is regulated by remodeling, with OCs resorbing old or damaged bone while OBs forming new bone [5]. This balance is essential for maintaining bone functions, including mechanical support for the body and serving as a calcium and phosphate reservoir [6]. When the homeostasis between the formation and the resorption is disrupted, abnormalities in the bone architecture may occur, with a reduction in bone mass, an increase in bone fragility, and a risk of bone fractures. This could lead to osteoporosis, the most common metabolic disease worldwide, particularly affecting the elderly and post-menopausal women [7, 8]. This condition affects approximately 200 million women globally [9]. It is estimated that one in three women and one in five men over the age of 50 will experience osteoporotic fractures. According to the World Health Organization [10], by 2025, the elderly population is projected to be 1.2 billion, leading to an enormous rise in healthcare and social support costs. Furthermore, by 2050, the population aged 60 years and older will double, reaching the number of 2.1 billion.

Among others, the endocannabinoid system (ECS) is a potential therapeutic target for osteoporosis [11]. The ECS is a family of ligands and G-protein-coupled receptors that regulate intracellular kinases to influence transcription. One of its receptors, cannabinoid receptor 1 (CB1), is expressed in vitro by OBs and OCs [12, 13]. However, the specific role of ECS signaling in bone development and the regulation of the activity of OBs and OCs remains unclear.

Numerous environmental factors, including the contaminant of emerging concern per- and polyfluoroalkyl substances (PFAS), were recently observed to adversely affect bone health. PFAS are a broad class of synthetic chemicals mainly used in a wide range of industrial processes. They are also found in many consumer products because of their unique hydrophobic and lipophobic properties and resistance to high temperatures. Nowadays, these compounds have attracted public health concern because their peculiar physio-chemical properties (resistance to chemical, physical, and metabolic degradation) make them highly persistent in the environment, with a high bioaccumulation capacity and a very long half-life [14]. Human exposure to PFAS is due to inhalation of contaminated dust and consuming contaminated food and water [15]. Due to their ability to disrupt hormonal balance and endocrine regulation, PFAS are also considered endocrine-disrupting chemicals (EDCs) [16]. Notably, PFAS have been observed to interfere with pathways involved in osteoblast formation and differentiation in vivo and 2D in vitro cultures [17, 18], although the mechanisms remain still unclear. Furthermore, recent studies have explored the relationship between PFAS exposure and bone-related outcomes, including osteoporosis and a higher risk of fractures [19–21]. Interestingly, EDCs exposure can also impact the ECS, and, given its role in maintaining bone homeostasis and contributing to ECM structure and formation, one mechanism of action of PFAS toxicity at the bone level could be mediated by ECS alteration. One of the most prevalent PFAS in the environment and human serum is perfluorooctanoic acid (PFOA) [22]. Over the last decade, this pollutant has garnered global attention due to its potential human hazards on cellular, genetic, neurological, cardiometabolic, reproductive, immunological, and cancer-related pathways [23]. Furthermore, it has been shown that exposure to this substance may directly influence the differentiation of mesenchymal stem cells (MSCs), leading to increased production of reactive oxygen species (ROS) in cells, negatively affecting the osteoblast mineralization process. [24–26]. Exposure to EDCs, such as Bisphenol A (BPA) or perfluoroctane sulfonic acid (PFOS) and PFOA, has been shown to impact MSCs’ proliferation and self-renewal capacity, inhibit osteogenesis, induce adipogenesis, increase oxidative stress, and promote a pro-inflammatory state. Collectively, these alterations impair the ability of MSCs to differentiate into appropriate lineages [26–28].

The present study used the hFOB1.19 cell line characterized by a multilineage differentiation potential [29]. This line demonstrates high osteoblastic activity, characterized by elevated alkaline phosphatase (ALP) levels and robust OCN expression after differentiative stimulus. They can also spontaneously mineralize the ECM, leading to the formation of calcified nodules. Furthermore, they exhibit low estrogen receptor expression, making them valuable for studying hormonal responses in human osteoblasts [30]. Spheroid culture obtained from this cell line has been studied regarding formation, aggregation, and suitability for microenvironmental research [29, 31].

The leading hypothesis of this study is that PFOA could affect osteoblast homeostasis differently during proliferative or differentiation phases, also impacting ECM deposition by interfering with oxidative stress defense, collagen degradation, and CB1 level alteration. 2D and 3D osteoblast cultures were used to test this hypothesis: the 2D model allows for the assessment of the impact of PFOA during the proliferative phase, while the 3D model provides insights into its effects during the differentiation phase.

## Results

### Effects of PFOA on the cell viability of 2D and 3D hFOB1.19 culture

The viability of hFOB1.19 during the proliferation phase was analyzed using an MTT assay in the case of 2D culture. Cells treated with 50 μM PFOA showed an average viability of 61.53% (*p* < 0.01), while those treated with 100 μM PFOA showed an average viability of 30.25% (*p* < 0.001), both indicating a significant reduction compared to the control. Consequently, these two concentrations were excluded from further analysis, and subsequent experiments were performed using 0.1, 1, and 10 μM PFOA (Supplementary Fig. S1a). In the 3D spheroid model, calcein/PI staining showed that exposure to 0.1 and 1 μM PFOA for 2 days caused a modest but statistically significant decrease in spheroid viability compared to control (~80%, *p* < 0.05). In contrast, higher concentrations (10 μM, 100 μM, and 1 mM) induced a more pronounced reduction in viability compared to control, which was highly significant (*p* < 0.01) (Supplementary Fig. S1b, d). When 3D hFOB1.19 were treated with PFOA for 5 days, cell viability significantly decreased in all treated groups compared to the CTRL group, identifying 1 mM PFOA as the DL50 (Supplementary Fig. S1c, e). As a result, the 1 mM PFOA group was excluded from further analysis. Overall, both 2D and 3D assays consistently show a dose-dependent decrease in cell viability, with higher PFOA concentrations exerting stronger cytotoxic effects.

### Impact of PFOA on 3D hFOB1.19 morphology

**Fig. 1:**
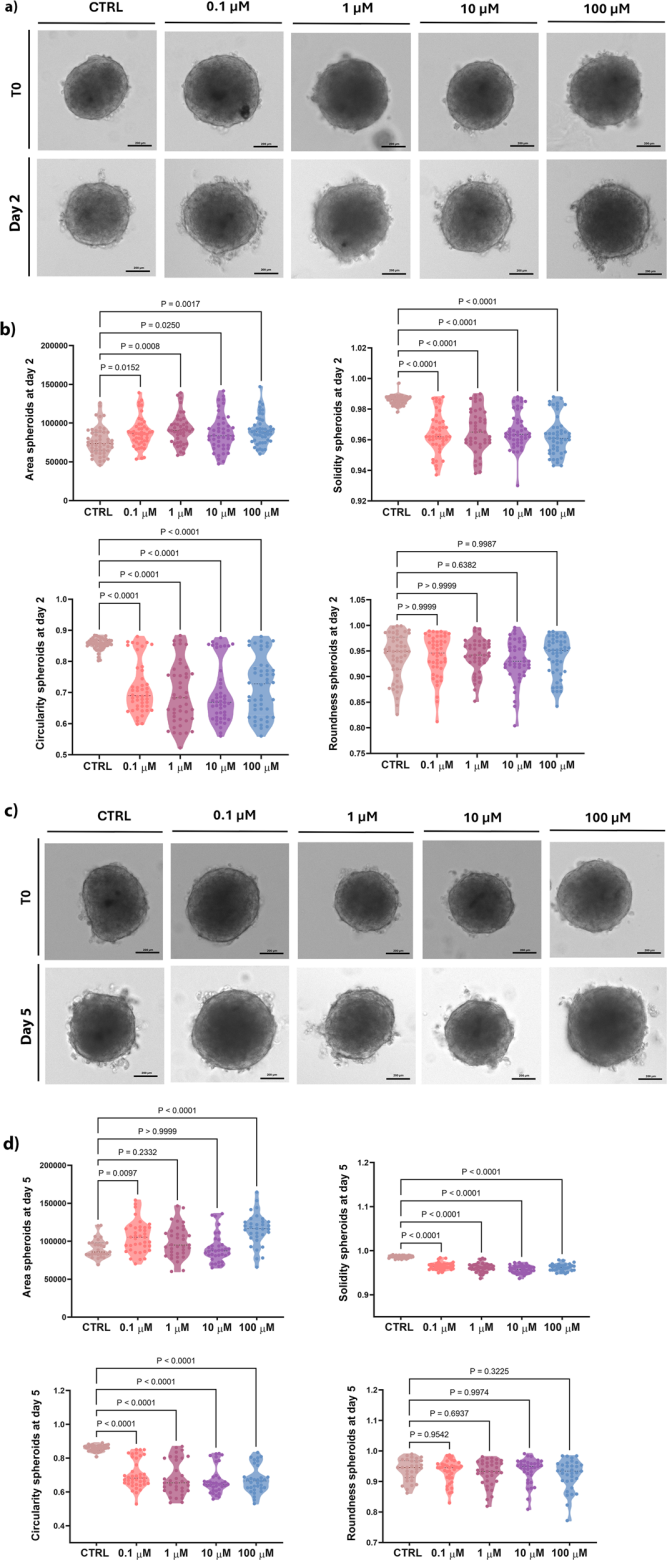
Morphometric analysis of 3D hFOB1.19 under differentiation. **a**, **c** Representative brightfield images of hFOB1.19 spheroids after 2 and 5 days of exposure to PFOA, respectively. Spheroids exposed to 0.02% of vehicle (DMSO) were used as the control. Images were obtained using the Light Lionheart FX Microscope. Scale bar = 200 μm (magnification 10×). **b**, **d** Morphological parameters of hFOB1.19 spheroids after 2 and 5 days of exposure to PFOA, respectively. ImageJ software determined area, solidity, circularity, and roundness after 2 and 5 days of exposure to different PFOA concentrations (*n* = 31–40), individual biological replicates. *P* values indicate the results of comparisons between each PFOA-treated group and the control group. Values of *p* < 0.05 are considered statistically significant.

To monitor the growth of spheroids exposed to different PFOA concentrations, parameters such as the area, diameter, solidity, roundness, and circularity were analyzed after 2- and 5-day exposure. Morphological changes during spheroid formation progressed through distinct timing were observed: after 2 days of treatment, circularity and solidity significantly decreased after exposure to all PFOA concentrations (Fig. 1a, b). Moreover, the measurements revealed a statistically significant increase in the area of spheroids exposed to 0.1, 1, 10, and 100 μM PFOA (Fig. 1b). After 5 days of exposure, spheroids treated with 0.1 and 100 μM PFOA showed a statistically significant increase in their area and a significant decrease in circularity and solidity measurements. Conversely, spheroids exposed to 1 and 10 μM PFOA exhibited a statistically significant reduction in circularity and solidity (Fig. 1c, d), while their area remained unchanged. In summary, PFOA exposure induced dose- and time-dependent morphological alterations in bone spheroids, characterized by increased area and reduced solidity and circularity, indicating impaired spheroid architecture.

### The effects of PFOA on calcium deposition during bone cell proliferation and differentiation

**Fig. 2:**
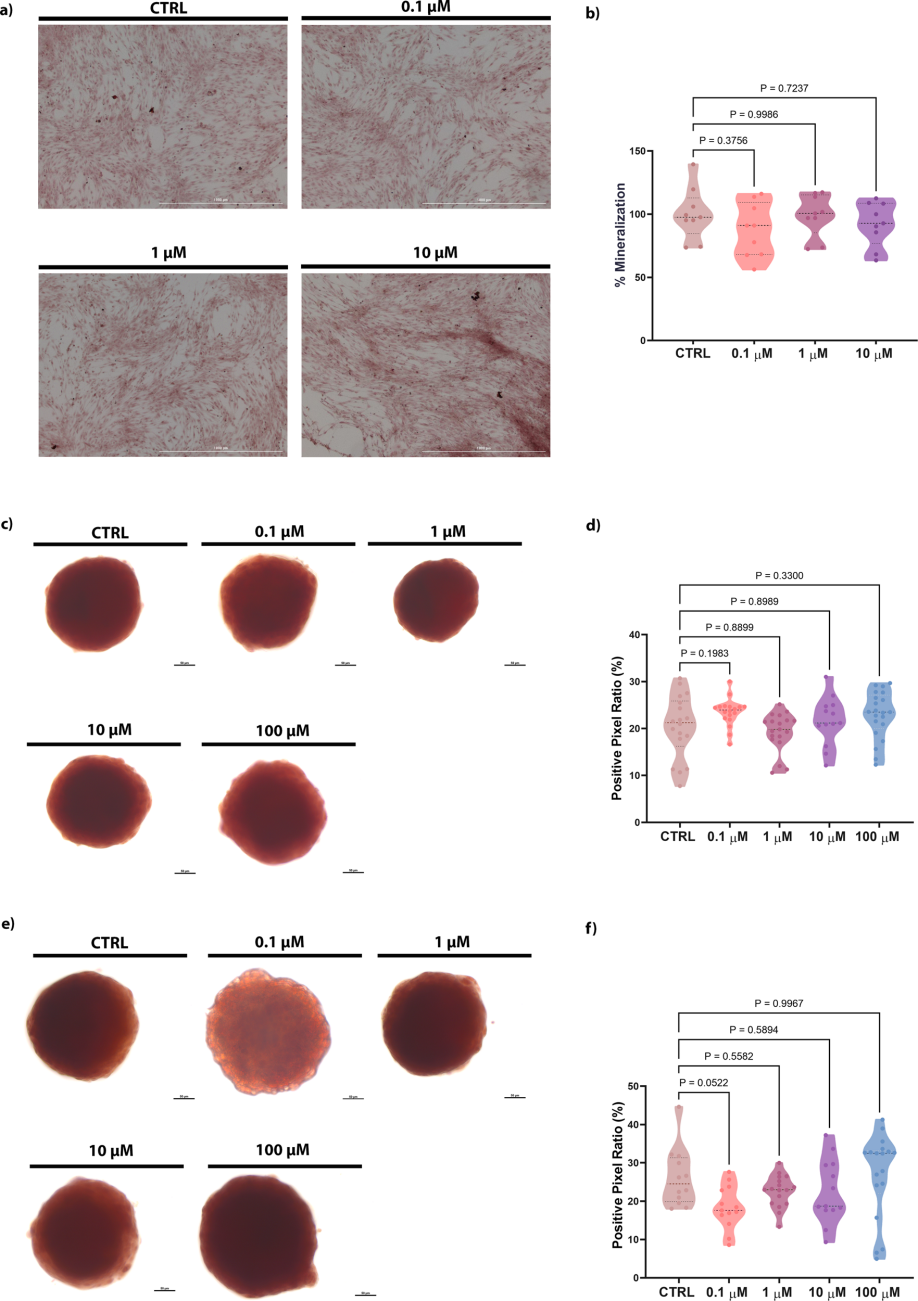
Assessment of calcium deposition on bone cells (2D and 3D models) following PFOA exposure. **a** Representative images of the control group and experimental groups treated with 0.1, 1, 10, 100 μM of PFOA; Scale bar = 1000 μM. Images captured using Light Lionheart FX Microscope (20×). **b** quantitative analysis of calcium deposition of all groups by absorbance measurement at 550 nm. Asterisks indicate the statistically significant data of the experimental groups (*n* = 9) compared with the control group (one-way ANOVA). **c** Alizarin red staining of spheroids treated with increasing concentrations of PFOA after 2 days of exposure; Scale bar = 200 μm. Images were captured using ZEISS Axio Imager A.2 (Zeiss, Oberkochen, Germany) with an Axiocam 503 camera (20×). **d** Quantitative analysis of the percentage of matrix mineralization in different treatment groups compared to the control group after 2 days of exposure (*n* = 15 individual biological replicates). **e** Alizarin red staining of spheroids treated with increasing concentrations of PFOA after 5 days of exposure; Scale bar = 200 μm. Images captured using ZEISS Axio Imager A.2 (Zeiss, Oberkochen, Germany) with an Axiocam 503 camera (20×). **f** Quantitative analysis of the percentage of ECM mineralization in different treatment groups compared to the control group after 5 days of exposure (*n* = 15 individual biological replicates). *P* values indicate the results of comparisons between each PFOA-treated group and the control group. Values of *p* < 0.05 are considered statistically significant.

Alizarin red staining was performed to evaluate the production and deposition of calcium nodules in bone cells treated with PFOA during different exposure windows. The exposure to the toxicant during the proliferative phase did not show any significant differences in the calcified deposits compared to the control group (Fig. 2a, b). Similar results were observed when exposing 3D hFOB1.19 (differentiation phase) to a 2-day treatment of PFOA (Fig. 2c, d). However, after exposing spheroids to 0.1 μM PFOA for 5 days, a decrease in the mineralization percentage was observed (*p* = 0.052) (Fig. 2e, f). Taken together, these findings indicate that PFOA exposure during proliferation does not markedly affect calcium deposition, whereas prolonged low-dose exposure during osteoblast differentiation may impair mineralization.

### The effects of PFOA exposure on osteogenesis during the proliferation phase

ALP (involved in the formation of ECM) protein levels and cytochemical staining were performed. The analysis showed that the protein level of ALP (78 kDa) and the % of positive-ALP area did not change after PFOA exposure during proliferation (Supplementary Fig. S2a, b). Further, the level of the transcription factor RUNX2 [32] was assessed by Western Blotting. The analysis of this transcription factor, essential for OB differentiation, did not show statistically significant differences after exposing bone cells to all PFOA concentrations. (Supplementary Fig. S2c).

Regarding PFOA epigenotoxic action during cell proliferation, two epigenetic marks, the acetylation of lysine 9 in histone 3 (H3K9ac) and lysine 12 in histone 4 (H4K12ac), were analyzed. The immunocytochemistry analysis revealed that the acetylation levels in these two histones were not altered by PFOA exposure in 2D culture (Supplementary Figs. S2d, e). Overall, PFOA exposure during the proliferation phase did not significantly affect osteogenic differentiation markers or epigenetic modifications in osteoblasts.

### The effects of PFOA exposure on bone oxidative stress

**Fig. 3:**
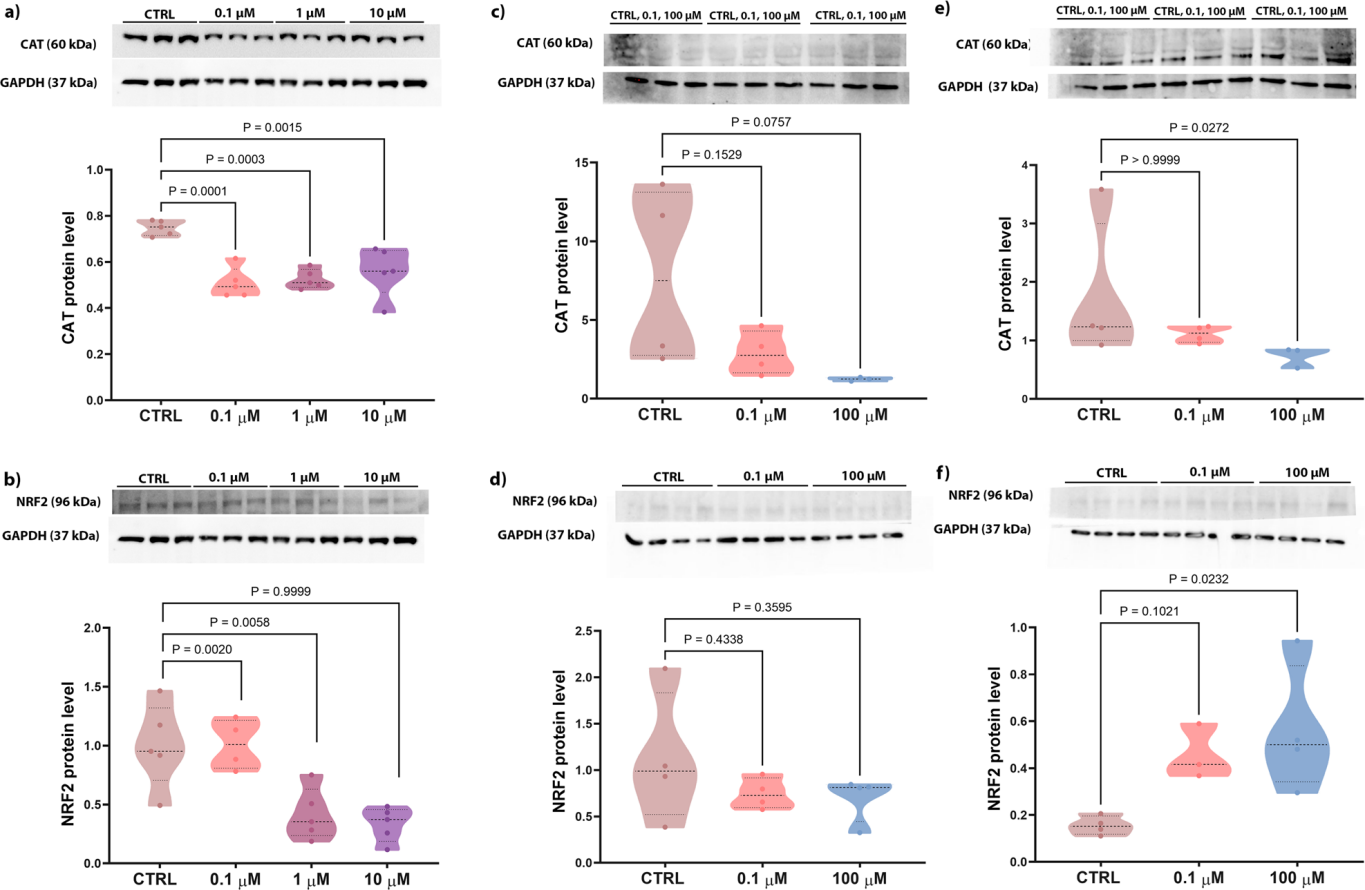
Analysis of oxidative stress markers in 2D and 3D osteoblast culture after PFOA exposure. Western blot analysis of oxidative stress marker expression, **a** (60 kDa) CAT and **b** (96 kDa) NRF2 for 2D (*n* = 5 biological replicates). **c**–**e** CAT levels in hFOB1.19 spheroids exposed to different PFOA concentrations for 2 days (**c**) and 5 days (**e**). NRF2 levels in hFOB1.19 spheroids exposed to different PFOA concentrations for 2 days (**d**) and 5 days (**f**) (*n* = 3–4 individual biological replicates). *P* values indicate the results of comparisons between each PFOA-treated group and the control group. Values of *p* < 0.05 are considered statistically significant.

Two proteins involved in oxidative stress were analyzed: CAT (catalyzing the breakdown of hydrogen peroxide to water and oxygen) and NRF2 (a nuclear erythroid 2-related factor regulating the expression of antioxidant proteins). In the 2D model, CAT protein levels significantly decreased in cells exposed to all doses of PFOA compared to the CTRL group (Fig. 3a), while NFR2 protein levels decreased in cells exposed to 1 and 10 µM PFOA (Fig. 3b). As for the 3D hFOB1.19 model, after 2 days of treatment, CAT levels were slightly lower after being exposed to 100 μM PFOA (*p* = 0.07) (Fig. 3c), with this decrease becoming significant after 5 days of exposure (Fig. 3e). Simultaneously, a statistically significant increase in NRF2 protein levels was observed in the spheroids exposed to 100 µM PFOA for 5 days, but not earlier (Fig. 3d, f). Taken together, the findings show that PFOA disrupts bone oxidative balance by reducing CAT levels at all doses and altering NRF2 regulation in a time- and concentration-dependent manner, thereby impairing antioxidant defense mechanisms.

### Effects of PFOA on bone extracellular matrix

**Fig. 4:**
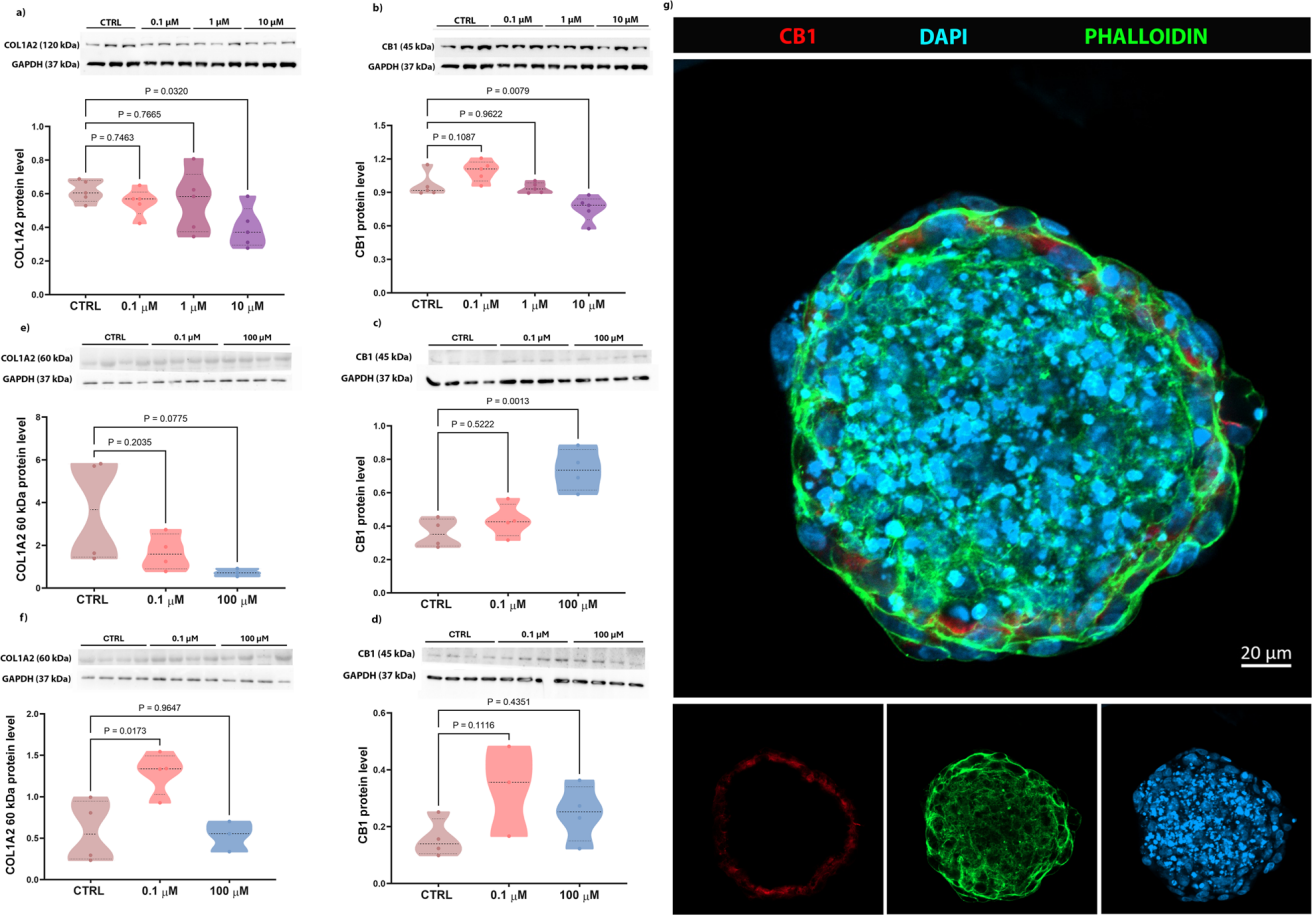
COL1A2 and CB1 protein levels in 2D and 3D osteoblast cultures. Western blot analysis of COL1A2 and CB1 levels. **a**, **b** (120 kDa) COL1A2 and (53 kDa) CB1 respectively for 2D (*n* = 5 individual biological replicates). **c**–**e** Degraded COL1A2 (60 kDa) levels in hFOB1.19 spheroids exposed to different PFOA concentrations for 2 and 5 days, respectively. **d**, **f** CB1 levels in hFOB1.19 spheroids exposed to different PFOA concentrations for 2 and 5 days, respectively (*n* = 3–4 individual biological replicates). *P* values indicate the results of comparisons between each PFOA-treated group and the control group. Values of *p* < 0.05 are considered statistically significant. **g** Localization of CB1 on the osteoblast membrane on the spheroid surface. Scale bar: 20 μm. Image captured using confocal microscope LSM 800 (Zeiss) (40×).

The levels of COL1A2 (the primary organic component of the ECM that provides bone elasticity) and the CB1 receptor (a protein potentially involved in collagen deposition) were analyzed by Western Blotting. In 2D cultures, COL1A2 protein levels were significantly reduced in cells treated with 10 µM PFOA compared to the CTRL group (Fig. 4a). Similarly, in the same group, CB1 levels were significantly reduced (Fig. 4b). Interestingly, in bone spheroids, exposure to PFOA led to a significant increase of the CB1 levels in the group treated with 100 µM PFOA after 2 days, while after 5 days of exposure (Fig. 4c, d) the levels were similar to control. As for the 0.1 µM PFOA dose, only 3 replicates were quantified (Fig. 4d). Additionally, the analysis of the degraded form of COL1A2 [33] in both PFOA-treated groups, after 2 days of exposure, showed lower levels more relevant at the highest dose (*p* = 0.07). After 5 days of exposure, degraded COL1A2 significantly increased when exposed to 0.1 µM PFOA concentration (Fig. 4e, f). In summary, these results suggest that PFOA may primarily act on the ECM through collagen-related pathways, with CB1 (Fig. 4g) potentially involved, and that its impact on these two protein levels varies depending on dose, exposure time, and culture model.

## Discussion

In this study, a dual approach to gain new insights into the impact and toxicity of PFOA during the proliferation and differentiation phases of human fetal osteoblasts was performed. A 2D culture system was used to study the effects of PFOA on human fetal osteoblasts during the proliferative phase, while a 3D osteoblast spheroid was used during osteoblast differentiation. Assessing the differences between 2D and 3D in vitro models is essential for understanding the complexity of bone function.

It is known that exposure to PFOA could suppress osteogenesis characterized by decreased mRNA expression of OB-specific genes, ALP activity, and calcium deposition [17, 34]. In the present study, the exposure from 0.1 to 10 μM PFOA during the proliferative phase did not directly impact the level of RUNX2, the key regulator of osteoblast differentiation that controls transcription factors like SP7 and bone matrix protein genes crucial for bone formation [35, 36]. Similarly, PFOA did not affect the acetylation levels of H3K9ac and H4K12ac. These significant epigenetic modifications play a pivotal role in activating the transcription of osteogenic genes such as osteoprotegerin and alkaline phosphatase *(ALPL)* during MSCs’ osteogenic differentiation [37].

Oxidative stress has been mainly linked to decreased bone formation by altering the production of key osteogenesis-related factors, such as RUNX2 [38–40]. Previous studies have reported that PFOA could induce oxidative stress [41–43]. Our results in 2D revealed a significant decrease in CAT levels, which are responsible for defense against oxidative stress, after exposure to all three doses of PFOA. PFOA exposure could affect the activity of this enzyme due to its ability to bind CAT, as suggested by Xu and colleagues [44]. PFOA compromises the antioxidant capacity of cells also by interfering with NRF2 protein levels. The NRF2 downregulation disrupted the antioxidant system, preventing the activation of defense mechanisms. This result is in accordance with previous studies showing that PFOA exerts its toxicity by inhibiting antioxidant enzyme function through the downregulation of *Nrf2* gene expression in the mouse testis and intestine [45, 46]. Accordingly, the significant downregulation of CAT and NRF2 protein levels observed in our study indicates that PFOA damages osteoblasts during the proliferation phase by impairing their ability to mount an appropriate response to oxidative stress. This aligns with previous studies showing that PFOA and other PFAS induce oxidative stress by altering the expression, translation, and activity of antioxidant enzymes in different cells [43, 47–49]. Similarly, 3D hFOB1.19 exposed to 100 µM PFOA exhibited a slight decrease in CAT levels after 2 days, which significantly decreased after 5 days of exposure. Notably, within this time frame, spheroids exposed to 100 µM PFOA for 5 days exhibited a significant increase in NRF2 levels compared to the control group, indicating an enhanced protective response mediated by NRF2 as previously demonstrated [46, 47]. This study also aligns with the findings by Sun et al. [50], where neuroblastoma cells exposed to PFOS (0, 10, 50, 100, 200 µM) for 48 h showed activation of the NRF2 signaling pathway in a dose-dependent manner, followed by ROS accumulation. Therefore, based on our results, we suggest that PFOA induces oxidative stress and triggers a defense response in spheroids during early differentiation, particularly at the highest dose. In addition, we focused on the effects of PFOA on 3D morphometric parameters. The impact of PFOA on the 3D hFOB1.19 model evidenced structural changes. This indicates that the contaminant affects their architectural development, pointing to changes in osteoblastic markers involved in ECM deposition, as also previously suggested [51]. To investigate whether PFOA affects ECM mineralization, we analyzed the Ca^2+^ deposition levels in both 2D and 3D models. Extracellular Ca^2+^ deposition is critical during osteoblast proliferation [50] as well as differentiation, specifically in matrix mineralization. The Alizarin red staining in the spheroids exposed to PFOA for 5 days revealed a significant decrease in calcium nodule deposition at 0.1 μM, no effect at intermediate concentrations, and an apparent increase at 100 μM, which are indicative of a non-monotonic dose-response, where the effect does not consistently increase or decrease with dose, but instead shows U-shaped or inverted U-shaped curves, meaning low doses can cause different or stronger effects than higher doses [52, 53]. Exposure to PFOA during proliferation in the 2D model did not alter ALP activity/level, which is essential for liberating inorganic phosphate necessary for further bone mineralization [54]. Likewise, Koskela and colleagues [55] reported no significant changes in ALP activity following exposure to PFAS during later stages of osteogenic differentiation (after 3 weeks) in bone marrow-derived MSCs from patients operated for hip osteoarthrosis.

Some studies have focused on the role of CB1 in osteoblast differentiation, particularly in mineralization and ALP production [56, 57]. ECM formation depends on the mineralization process and the secretion of COL1, which appears to be a predictive marker for bone spheroid formation alongside ALP [58, 59]. Moreover, evidence on the role of CB1 in modulating collagen deposition have been described [60, 61]. CB1 inactivation has been reported to reduce fibroblast activation and collagen deposition in vitro [62], and, therefore, targeting CB1 may offer a strategy for monitoring collagen production as suggested by Wang and colleagues [63]. We investigated the impact of PFOA treatment on CB1 and, subsequently, on COL1A2 levels. In 2D culture, our results showed reduced CB1 and COL1A2 protein levels in response to 10 µM PFOA. This aligns with the findings of Yang et al. [17], who reported a significant downregulation of *COL1A1* expression at the highest dose of PFOA tested (25 µM) [17]. Similarly, in the 3D model, we observed an intriguing interplay between CB1 and degraded COL1A2 (60 kDa). Notably, after 2 days of contaminant exposure, CB1 overexpression in the 100 µM group corresponded to a lower amount of degraded collagen. Over 5 days, no changes in degraded collagen were observed in the 100 µM PFOA group, probably due to the high CB1 levels found at 2 days of exposure. Interestingly, the 0.1 µM PFOA evidenced a significant increase in degraded collagen unrelated to CB1 levels, suggesting additional players in COL1A2 degradation affected by PFOA. These results indicate that CB1 may be involved in osteoblast collagen production, as reported in fibroblasts [64], particularly during early differentiation stages. However, the precise mechanisms linking CB1 and COL1A2 and their functional activity remain unclear and require further investigation.

Our findings demonstrate that PFOA exerts phase and dose-dependent effects on osteoblast function and ECM organization. In 2D cultures, prolonged PFOA exposure primarily impaired antioxidant defense without modifying phosphate and calcium deposition. Conversely, in 3D spheroids, PFOA disrupted spheroid morphology and calcium ECM deposition in a dose- and time-specific manner. In both 2D and 3D models, lower concentrations of PFOA (0.1–1 μM) primarily disrupted bone homeostasis by impairing mineralization and altering protein expression, respectively, whereas higher concentrations (10–100 μM) exerted stronger cytotoxic effects by reducing cell viability and compromising antioxidant defenses. This distinction underscores the dose-dependent and multifaceted adverse impact of PFOA on bone cell function. Collectively, our results emphasize the necessity of evaluating both low- and high-dose exposures in complementary 2D and 3D bone models, as the 2D system captures early cellular responses, while the 3D system better recapitulates the in vivo microenvironment and ECM organization.

## Materials and methods

### hFOB1.19 cell culture

The human osteoblast cell line hFOB1.19 (ATCC CRL-11372) was obtained from the American Type Culture Collection (ATCC, USA) in March 2021. The cell line was originally established by transfection of limb tissue obtained from a spontaneous miscarriage with the temperature-sensitive expression vector pUCSVtsA58 and the neomycin resistance expression vector pSV2-neo, and clones were selected in the presence of 0.6 mg/mL G418. The identity of the cell line was authenticated by short tandem repeat profiling and confirmed to be free of mycoplasma contamination by the company. Human fetal osteoblast cell line (hFOB1.19; CRL-3602) was cultured in a 1:1 mixture of Ham’s F12 Medium and Dulbecco’s Modified Eagle’s Medium (DMEM) supplemented with 2.5 mM L-glutamine and without phenol red (Life Technologies Limited, Paisley, UK). The medium was enriched with 0.3 mg/mL G418/Geneticin (Life Technologies Corporation, Grand Island, NY, USA) and 10% Fetal Bovine Serum (FBS; GIBCO). Cells were maintained at 34 °C in a humidified incubator with 5% CO_2_ following the conditions described by Sojan and collaborators [32]_._ Subculturing was performed at 80% confluence, and cells between passages 10 and 15 were used for all the subsequent experiments.

### hFOB1.19 spheroid formation

The 3D spheroid model was performed using the hanging drop technique, as described by Ryu and colleagues [65]. A cell suspension at a density of 2 × 10^3^ cells/25 µL per drop was prepared in DMEM and seeded onto a Petri dish lid. The plates were incubated at 34 °C and 5% CO_2_ for 3 days. After 3 days, the spheroids were transferred individually into a 96-well plate coated with 1% agarose to prevent adhesion to the bottom of the wells. After 2 days from the transfer into the 96-well plate, the spheroids were cultured in osteogenic medium (OM) composed by a 1:1 mixture of Ham’s F12 Medium and DMEM supplemented with 2.5 mM L-glutamine, 0.3 mg/ml of G418/Geneticin, 10% FBS, 50 μg/mL ascorbic acid (Sigma-Aldrich, St. Louis, MO, USA) and 7.5 mM β-glycerophosphate (Sigma-Aldrich, St. Louis, MO, USA). The cultures were maintained at 34 °C and 5% CO_2_ to stimulate mineralization and used for PFOA exposure for up to 10 days post-spheroid formation. Images were captured at the time points specified in the corresponding figures.

### PFOA treatments

In the cytotoxicity experiments, the tested range of PFOA was primarily between 0.1 and 1000 μM, selected based on the existing literature on PFOA levels in human serum (0.001 to 100 μM) in both environmental and occupational scenarios, mimicking real-life exposure conditions [17, 66, 67]. PFOA (95%, 171468-256, Sigma-Aldrich, St. Louis, MO, USA) was first dissolved in DMSO before being diluted in the culture medium. Cells were exposed to either a final concentration of 0.02% (v/v) DMSO (control group) or PFOA at concentrations of 0.1, 1, 10, 50, 100, and 1000 μM. In 2D culture, the exposure lasted for 6 days (considering the initial time frame observed during osteoblast differentiation after osteogenic induction) [68, 69], with medium changes performed halfway through the treatment. For 3D spheroids, treatment was initiated 2 days after their transfer into 96-well plates. The spheroids were exposed to the contaminant diluted in OM for 2 and 5 days, corresponding to the 7th and the 10th days of spheroid formation, respectively.

### MTT assay for 2D osteoblast culture

For 2D experiments, osteoblasts were seeded at a concentration of 2 × 10^4^ cells/well in 48-well plates. The MTT assay was performed on day 6 of treatment following the protocol described by Sojan and colleagues [32]. Absorbance was measured at 550 nm using a microplate reader (Synergy, Biotek, Winooski, VT, USA).

### LIVE/DEAD staining for osteoblast spheroids

Spheroid viability was evaluated using the Live/Dead Cell Imaging Kit (488/570) (ThermoFisher, R37601) on days 2 and 5 of treatment. Spheroids were incubated for 2 h at 34 °C, adding 25 μl of the staining solution, and then transferred into the μ-SLIDE 8-well-high Glass Bottom (Ibidi®, cat#80807) with the addition of 100 μl phosphate-buffered saline (PBS). Fluorescence images were acquired under a confocal microscope Nikon A1R, capturing approximately 50 z-stacks (1.4 μm per stack) per spheroid. Quantification was performed using ImageJ by thresholding each channel (green for calcein-AM, red for Propidium Iodide) and measuring the stained area. For analysis, 25 z-stacks per spheroid were quantified to calculate the percentage of the total area of live cells following the formula: %live cells = (area positive for calcein-AM (live cells)/area positive for calcein-AM (live cells) + area positive for PI (dead cells)) × 100%. Fluorescence images were acquired under identical confocal settings (laser power, gain, exposure time, pinhole) for all conditions.

### Monitoring the growth and morphology of spheroids

10× magnification images were captured using the Light LionHeart FX Automated Microscope after 2 and 5 days from the 5th day of spheroid formation, corresponding to 2-day and 5-day PFOA exposure periods. Images were analyzed using Fiji ImageJ software (https://imagej.net/downloads) to evaluate parameters such as area, roundness, solidity, and circularity. The roundness (calculated as 4 × area/(*π* × major_axis^2^)) measures how closely the shape of the 2D spheroid image approximates a perfect circle, reflecting the gross morphological features of the spheroids. Conversely, solidity serves as an indicator of the roughness and regularity of the spheroid surface. The circularity (Cir) is calculated as 4 × *π* × area/perimeter^2^ [70, 71].

### 2D alkaline phosphate staining

Cells were washed with DPBS to remove culture medium. They were then incubated with the freshly prepared staining mixture for ALP provided in the BCIP/NBT kit (Sigma-Aldrich, St. Louis, MO, USA) for 45 min at 37 °C in dark conditions following the kit protocol. To prevent light-induced degradation, incubation was performed covered with aluminum foil. The reaction was stopped by rinsing with PBS containing 20 mM EDTA, followed by two washes with deionized water (1 mL/well) and photographed using a Lionheart XF Automated Microscope (Biotek, Winooski, VT, USA).

### 2D and 3D alizarin red staining

Osteoblasts were seeded at a concentration of 1 × 10^5^ cells/mL in 12-well plates. After the treatments, cells were washed three times with PBS and fixed in 4% (w/v) paraformaldehyde (PFA) for 30 min at 4 °C. After fixation, cells were washed three times with Milli-Q water. Alizarin red staining was performed following the protocol previously described by Sojan et al. [32]. Images of 2D cultures were captured using the Light LionHeart FX Automated Microscope. The analysis of % mineralization was conducted by measuring absorbance values, which were subsequently converted into the percentage of mineralization relative to the control.

Spheroids on days 2 and 5 post-treatment were collected into tubes and fixed in 4% (w/v) PFA for 1 h at room temperature under shaking. After fixation, spheroids were washed three times with milli-Q water by centrifugating at 100 × *g* for 2 min. Staining was performed using the protocol mentioned above. Each spheroid was positioned on a concave glass slide for observation under a ZEISS Axio Imager A.2 (Zeiss, Oberkochen, Germany) with an Axiocam 503 camera (20×) in brightfield. Images were acquired at 3 focal planes for each spheroid. The staining intensity of alizarin red was quantified using Fiji ImageJ software [72]. The positive pixel ratio of each spheroid was calculated using the following formula: Positive Pixel Ratio = (number of positive pixels)/(number of pixels of the total area).

### Protein extraction and Western blotting

For protein extraction from the 2D culture, osteoblasts were seeded at 5 × 10^4^ cells/mL in 100 mm Petri dishes. After PFOA treatment, cells were washed with PBS 1X and collected. Then, the samples were centrifuged at 1000 × *g* for 5 min at 25 °C, and the supernatant was removed. The pellets were then kept at −80 °C until proceeding with protein extraction. Western blot was performed following the same protocol previously described by Sojan and colleagues [32].

In the 3D spheroid culture, 65 spheroids for each technical replicate were collected and washed with PBS 1X. Proteins were extracted using Hanna’s buffer containing 0.125 M Tris-HCl pH 7.5, 4% (w/v) SDS, 20% (v/v) glycerol, and 10% (v/v) β-mercaptoethanol, supplemented with 1:10 Protease Inhibitor Cocktail (Roche, Basel, Switzerland) and Anti-Phosphate Inhibitor Cocktail 1 mM (Sodium Orthovanadate, Sigma-Aldrich, cat#S6508 and Sodium Fluoride, Sigma-Aldrich, cat#7681-49-4). The protein extracts were incubated at 4 °C for 30 min and centrifuged at 12,000 × *g* for 15 min. The supernatant was collected, and proteins were run in SDS-PAGE gel (12% of acrylamide for the analysis of NRF2, CAT, and CB1, and 15% for the analysis of COL1A2). The proteins were transferred into a nitrocellulose membrane for immunoblotting, incubating for 2 h at 250 mA in transfer solution (25 mM Tris, 192 mM glycine, and 20% (v/v) ethanol) for the proteins with high molecular weight and for 90 min at 200 mA for the proteins with low molecular weight. To block non-specific sites, membranes were then incubated with 5% BSA in Tris-Buffered Saline with 0.1% Tween 20 (TBS-T), followed by overnight incubation at 4 °C with primary antibodies (described in Supplementary Table 1) in 5% BSA in TBS-T. Subsequently, the membranes were washed with TBS-T three times and incubated with secondary antibodies, anti-rabbit IgG-HRP and anti-mouse IgG-HRP conjugated with horseradish peroxidase (Sigma-Aldrich®, Milan, Italy), diluted 1:2500. Detection of antibody binding was carried out using Clarity Western ECL Substrate (Bio-Rad, Hercules, CA, USA). Images were acquired with the ChemiDoc XRS+ System (Bio-Rad Laboratories). Densitometric analysis was conducted using Fiji ImageJ software, and protein levels were normalized against GAPDH as indicated. The full and uncropped western blots are provided in the Supplemental Material.

### Immunocytochemistry and whole-mount immunostaining

For 2D culture, cells were seeded at a density of 7 × 10^4^ cells/well in 24-well plates on top of coverslips. After PFOA treatments, immunocytochemistry was performed following the protocol described by Escarda-Castro and colleagues [73]. Primary antibodies (described in Supplementary Table 1) were incubated in a blocking solution overnight at 4 °C. Following this, cells were washed three times with PBS and then incubated with a secondary antibody (Goat Anti-Rabbit IgG H&L, Alexa Fluor ® 488, 150077, Abcam, Cambridge, UK) diluted 1:500 in PBS for 60 min, protected from light. After three washes in PBS, cells were incubated with 1:100 TRITC-labeled phalloidin (Alexa Fluor Plus 647, A30107, 400X) in 1% BSA for 60 min and rewashed three times with PBS. Nuclei were counterstained with 1 µg/mL diamidino-2-phenylindole (DAPI) for 5 min. Eventually, coverslips with the prepared samples were mounted upside down on the slides using Vectashield mounting medium (Vector Laboratories Inc., Newark, CA, USA) and observed with a confocal microscope Nikon A1R.

A semi-quantitative analysis measured the corrected total cell fluorescence. This approach evaluated histone acetylation levels by quantifying the fluorescence intensity per unit of nuclear area. At least 100 nuclei per slide were analyzed under each condition to obtain reliable data. This method provides a normalized assessment of histone acetylation, allowing comparisons across different experimental groups. Histone acetylation levels were quantified as fluorescence intensity per nucleus area (at least 100 nuclei per slide were analyzed) on Fiji ImageJ Software.

For 3D culture, fixed spheroids were permeabilized with PBSFT (10% BSA, 1% Triton X-100 in PBS) for 1 h at 4 °C in agitation. CB1 was incubated overnight at 4 °C with agitation. On the second day, the sample was washed two times (5 min), three times (15 min), and six times (1 h) in PBSFT at 4 °C on a shaker, followed by incubation with the appropriate secondary antibodies, DAPI solution (DAPI 1:10000 in PBSFT), and labeled with phalloidin (Alexa Fluor 488, A12379). On the third day, the samples were washed two times (5 min) in PBSFT and two times (5 min) and three times (15 min) at room temperature in PBT solution (0.2% BSA + 0.2% Tryton X in PBS). Spheroids were kept in 30% glycerol in PBT. Confocal images were acquired with the confocal microscope LSM 800 (Zeiss).

### Statistical analysis

Statistical analyses for 2D cells and 3D spheroids were performed using Graphpad V8.0.1 (GraphPad Software, Inc., San Diego, CA, USA). The normality of the data was checked using Shapiro–Wilk test (*p* > 0.05). Differences between the control and treatment groups were analyzed using one-way ANOVA for parametric data, whereas, for non-parametric data, a Kruskal–Wallis test was applied. Values of *p* < 0.05 were considered statistically significant. Sample sizes were determined based on preliminary experiments to ensure adequate statistical analysis.

## Supplementary information

Supplementary Figures and Table

Uncropped Western Blots

Live Dead Quantification

## Data Availability

The datasets generated during and/or analyzed during the current study are available from the corresponding author on reasonable request.
