# Supplementary material for: Unveiling the toxic effects of perfluorooctanoic acid on osteoblast function and extracellular matrix deposition using 2D and 3D models
Source: Cell Death Discov. 2026 Jan 9;12:10. doi: 10.1038/s41420-025-02863-5 (PMC12789562; doi:10.1038/s41420-025-02863-5)
Supplement: Supplementary file 1 — Supplementary Figures and Table [file 41420_2025_2863_MOESM1_ESM.docx]

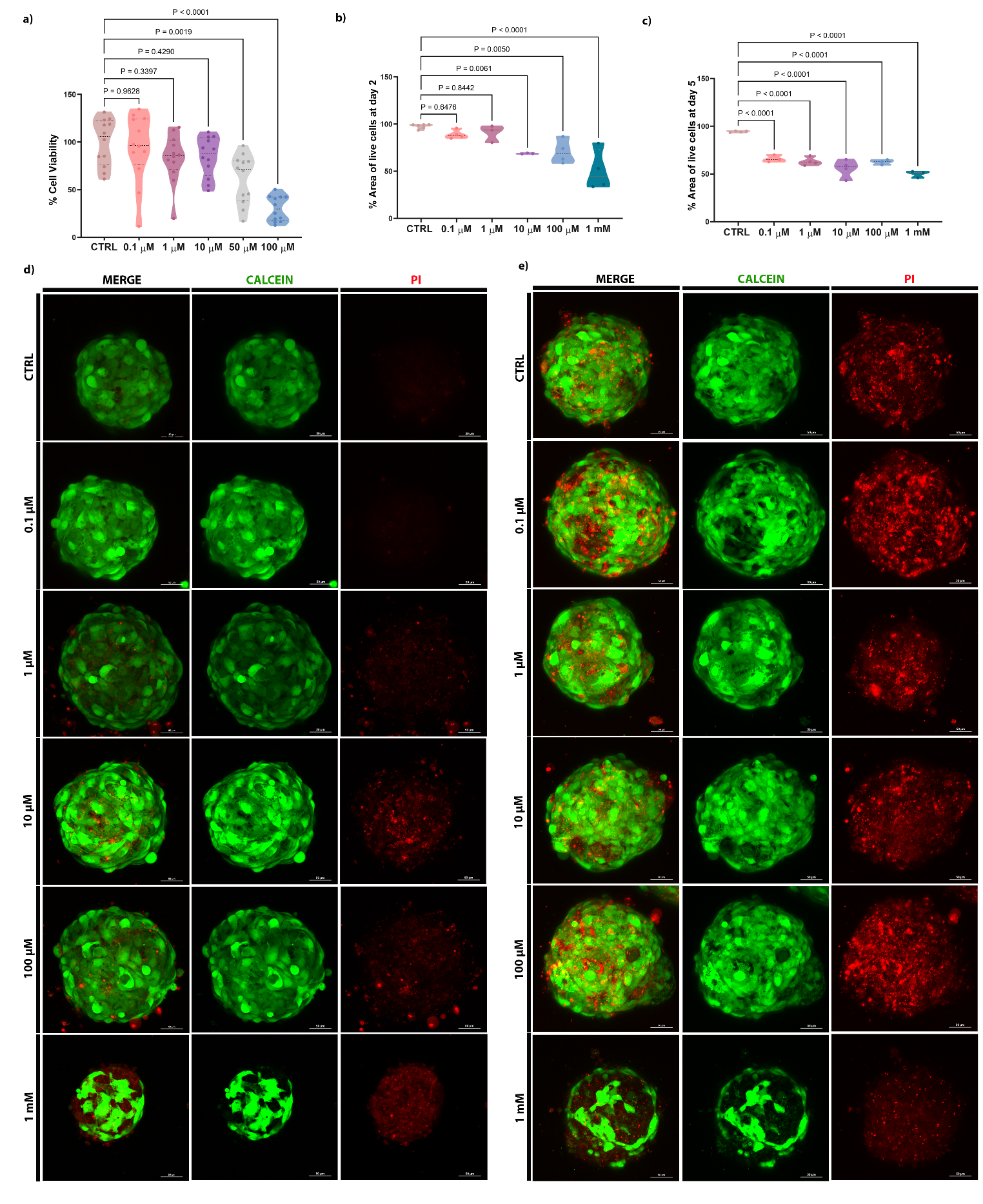


**Supplementary Figure 1. Cell viability of 2D and 3D hFOB1.19 cultures exposed to PFOA during proliferative and differentiation** **phases.**

**a)** Analysis of cell viability of hFOB1.19 2D culture after exposure to different concentrations of PFOA (n=12 independent biological replicates) by MTT assay. P-values indicate the results of comparisons between each PFOA-treated group and the control group. Values of p < 0.05 are considered statistically significant. **b-c)** Analysis of cell viability of hFOB1.19 spheroids after exposure to different concentrations of PFOA (n = 3-4 independent biological replicates) in two different time points by LIVE/DEAD staining. Quantification was performed by measuring the stained area in each channel using ImageJ and the percentage of live cells was then calculated as %live cells = (area positive for calcein-AM (live cells)/area positive for calcein-AM (live cells) + area positive for PI (dead cells)) x 100. P-values indicate the results of comparisons between each PFOA-treated group and the control group. Values of p < 0.05 are considered statistically significant. **d-e)** Maximum Intensity Projection (MIP) of representative confocal images showing of the LIVE/DEAD staining after 2 days (d) and 5 days (e) of PFOA treatment. Live cells were stained with calcein (green), while dead cells were stained with propidium iodide (PI) (red). Scale bar=50 μm.

**
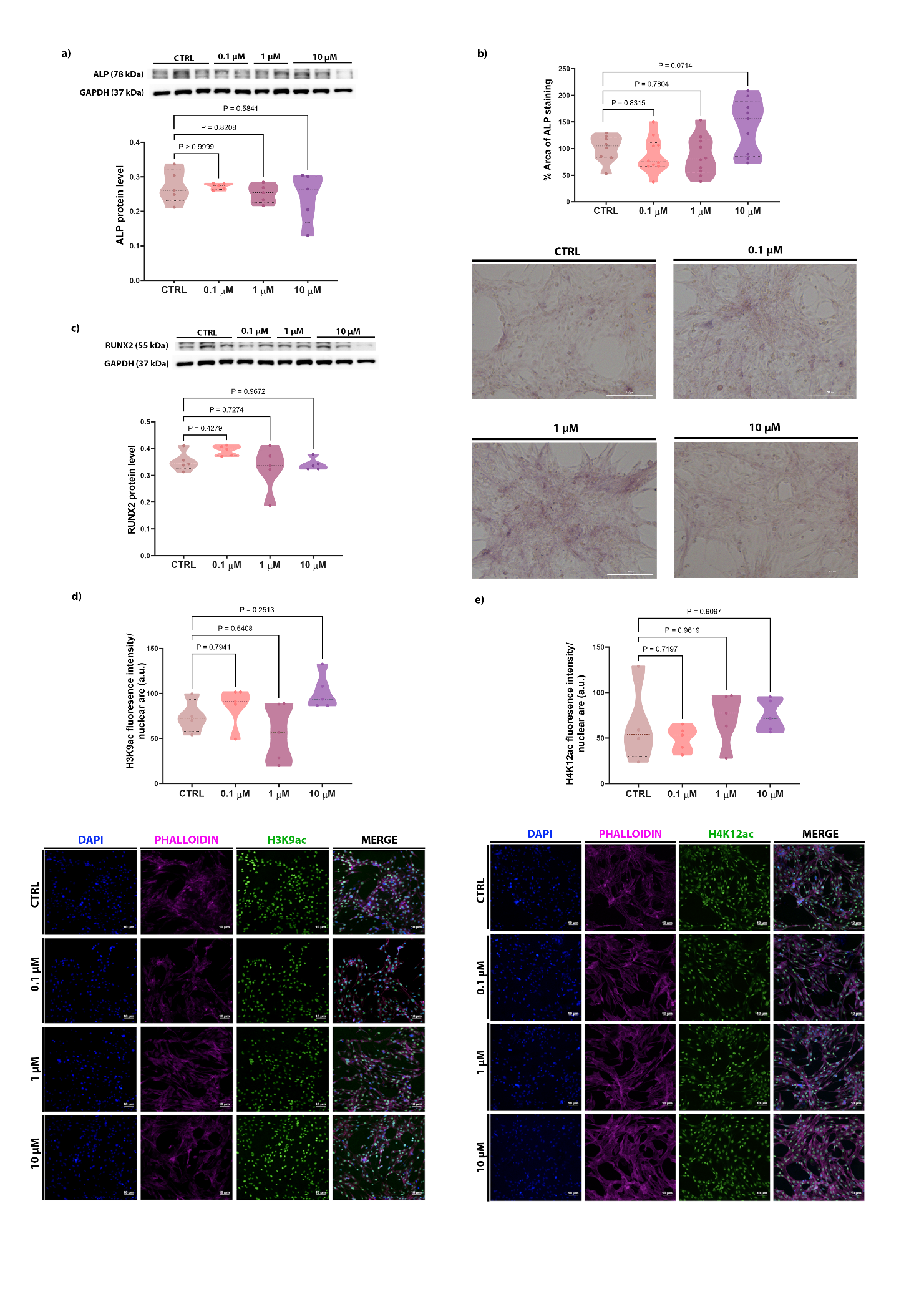
**

**Supplementary Figure S2. Osteogenic markers correlate with PFOA responsiveness in 2D osteoblast after 5 days of exposure.**

Analysis of osteogenic marker protein levels by western blot **a)** ALP 78 kDa **b)** ALP % positive area intensity measured using ImageJ and converted to % values over control. One-way ANOVA was used to analyze the difference between the groups and Tukey’s post hoc test was used for multiple comparisons among groups **c)** RUNX2 in hFOB1.19 cells exposed to different PFOA concentrations. P-values indicate the results of comparisons between each PFOA-treated group and the control group. Values of p < 0.05 are considered statistically significant. **d and e)** The violin blots represent the mean fluorescence intensity (MFI) profiles of H3K9ac and H4K12ac levels, respectively, in hFOB1.19 cells exposed to different PFOA concentrations (n = 5 individual biological replicates). Representative confocal images of hFOB1.19 cells immunostained for histone acetylation (green) and F-actin (purple); nuclei were counterstained with DAPI (blue). Scale bar: 10 µm.

**Table 1**. List of primary and secondary antibodies used in Western blotting (WB) and Immunofluorescence (IF) analysis.

| **Primary Antibody** | **Diluition of primary antibody** | **Diluition of secondary antibody** | **Origin** | **Experimental Analysis** | **Manufacturer primary antibodies** | **Catalogue number** |
| --- | --- | --- | --- | --- | --- | --- |
| anti-RUNX2 | 1:1000 | 1:10000 | Anti-rabbit | WB | Sigma-Aldrich | HPA022040-100UL |
| anti-ALP | 1:1000 | 1:30000 | Anti-mouse | WB | Abcam | ab126820 |
| anti-COL1A2 | 1:1000 | 1:10000 | Anti-rabbit | WB | Proteintech | 14695-1-AP |
| anti-NRF2 | 1:500 | 1:1000 | Anti-rabbit | WB | Cell Signaling  Technology | 12721 |
| Stress defense cocktail (anti-CAT, anti-SOD-1, anti-TRX) | 1:1000  1:500 | 1:10000 (2D)  1:2500 (3D) | Anti-rabbit | WB | Abcam | ab179843 |
| anti-CB1 | 1:1000 1:1000  1:100 | 1:30000 (2D) 1:2500 (3D) | Anti-rabbit | WB  IF | Abcam | ab259323 |
| anti-GAPDH | 1:10000 | 1:30000 (2D) | Anti-mouse | WB | Proteintech | 60004-1-Ig |
| anti-GAPDH | 1:1000 | 1:2500 (3D) | Anti-rabbit | WB | Cell Signaling  Technology | 2118 |
| Anti-H3K9ac | 1:200 | 1:500 | Anti-rabbit | IF | Abcam | ab10812 |
| Anti-H4K12ac | 1:200 | 1:500 | Anti-rabbit | IF | Abcam | ab46983 |
| **Secondary Antibody** | **Diluition of primary antibody** | **Diluition of secondary antibody** | **Origin** | **Experimental Analysis** | **Manufacturer primary antibodies** | **Catalogue number** |
| Goat Anti-Rabbit IgG H&L (Alexa Fluor® 488) |  | 1:400 | Anti-rabbit | IF | Abcam | ab150077 |
| Goat Anti-Mouse IgG H&L (Alexa Fluor® 647) Phalloidin |  | 1:400 | Anti-mouse | IF | Abcam | ab150115 |
| Anti-mouse |  | 1:30000 |  | WB 2D | Bethyl Laboratories | A90-116P |
| Anti-rabbit |  | 1:10000 |  | WB 2D | Invitrogen | 65-6120 |
| Anti-rabbit |  | 1:2500 |  | WB 3D | Sigma-Aldrich | A0545 |
