# Supplementary material for: Unveiling the toxic effects of perfluorooctanoic acid on osteoblast function and extracellular matrix deposition using 2D and 3D models
Source: Cell Death Discov. 2026 Jan 9;12:10. doi: 10.1038/s41420-025-02863-5 (PMC12789562; doi:10.1038/s41420-025-02863-5)

Supplemental Material – Original Western Blots

Relevant areas for cropped blots in the main and Supplementary figures are shown with dashed box

2D

Figure 3a

CTRL 0.1 μM 1 μM 10 μM

CAT 60 kDa

GAPDH 37 kDa

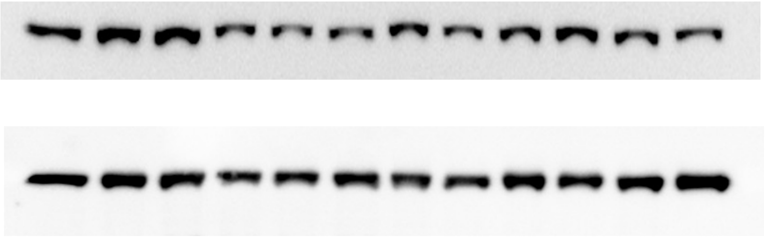

3D

Figure 3c

2 days

CTRL, 0.1 μM, 100 μM CTRL, 0.1 μM, 100 μM CTRL, 0.1 μM, 100 μM

CAT 60 kDa

GAPDH 37 kDa

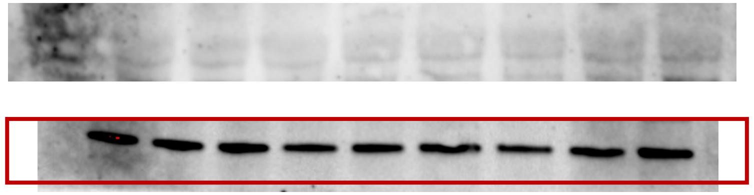

75 kDa

50 kDa

Figure 3e

5 days

CTRL, 0.1 μM, 100 μM CTRL, 0.1 μM, 100 μM CTRL, 0.1 μM, 100 μM

CAT 60 kDa

GAPDH 37 kDa

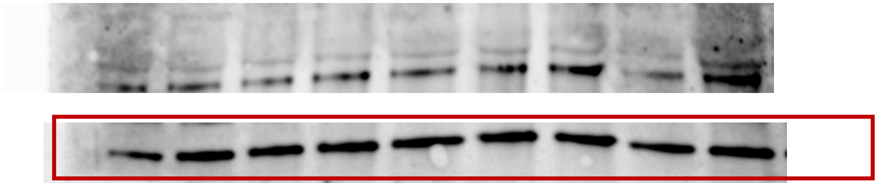

75 kDa

50 kDa

37 kDa

2D

Figure 3b

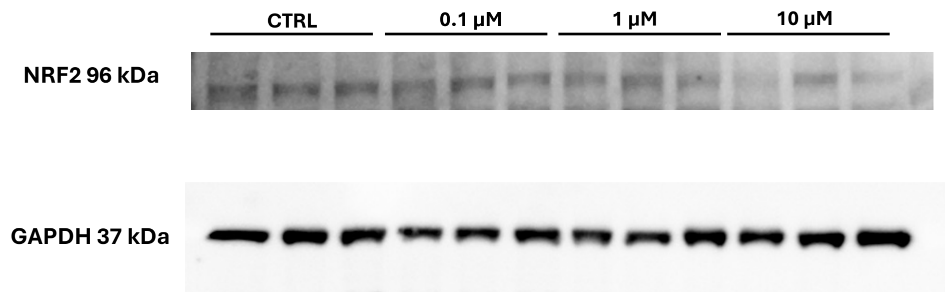

3D

Figure 3f

5 days

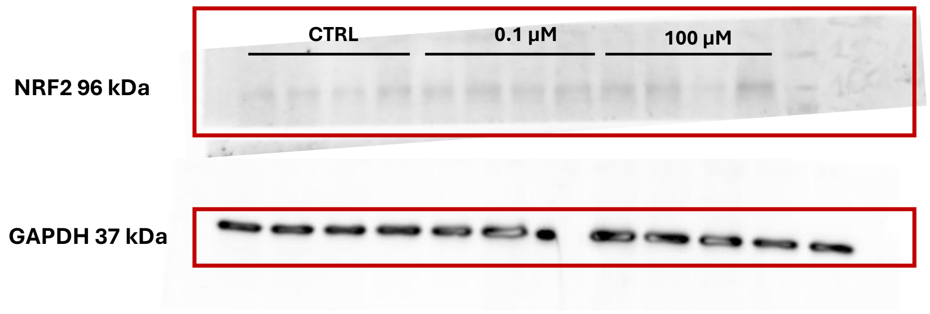

Figure 3d

2 days

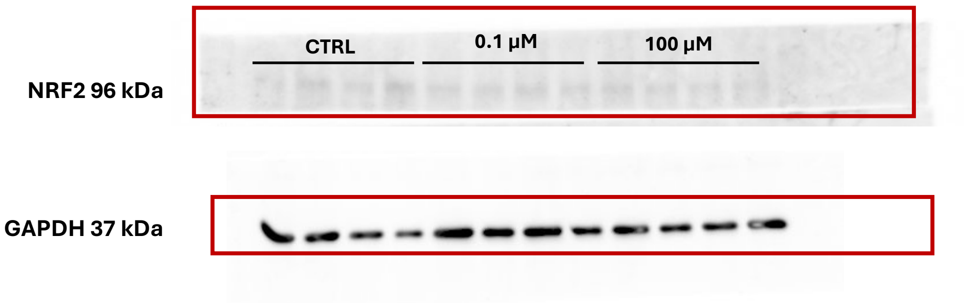

2D

Figure 4a

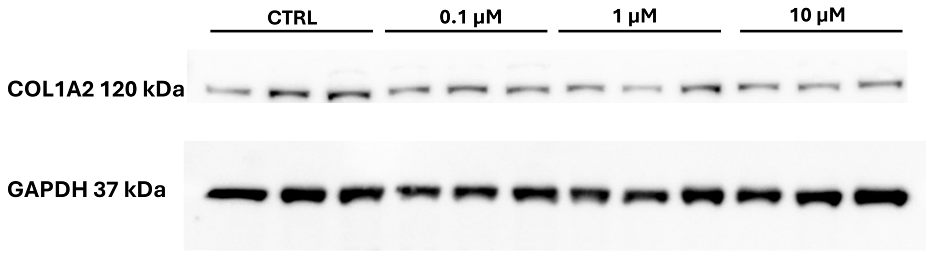

3D

2 days

Figure 4e

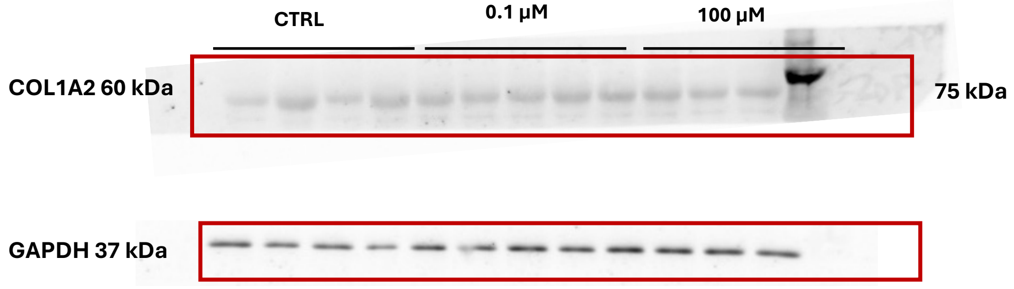

5 days

Figure 4f

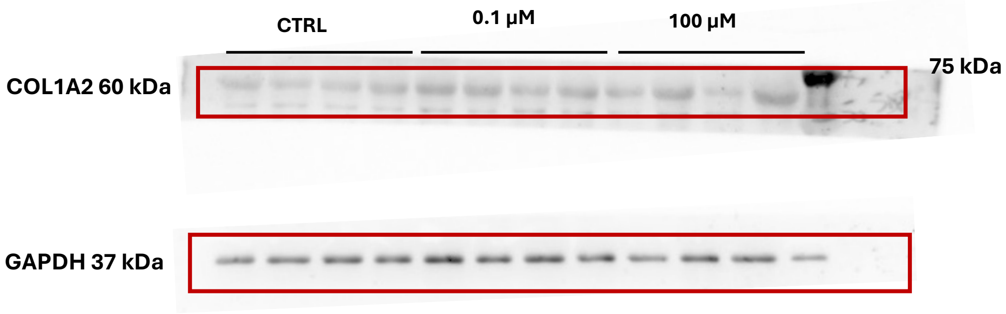

2D

3D

Figure 4b

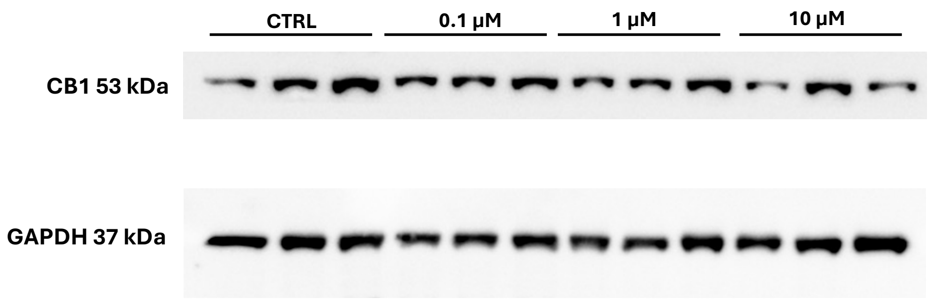

Figure 4d

5 days

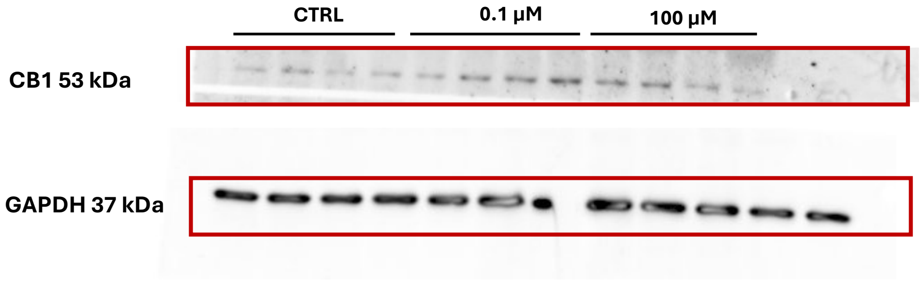

2 days

Figure 4c

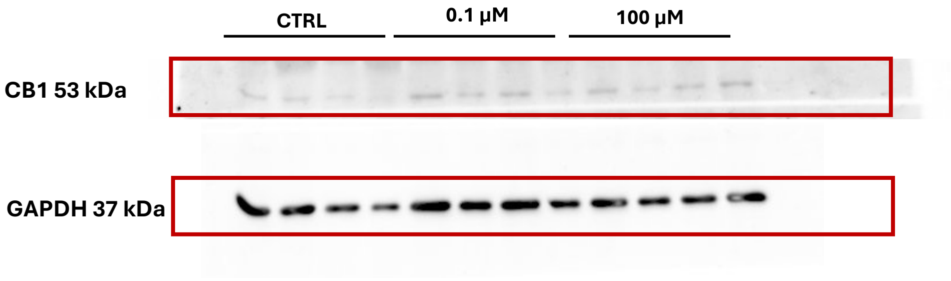

Figure S2a and S2c

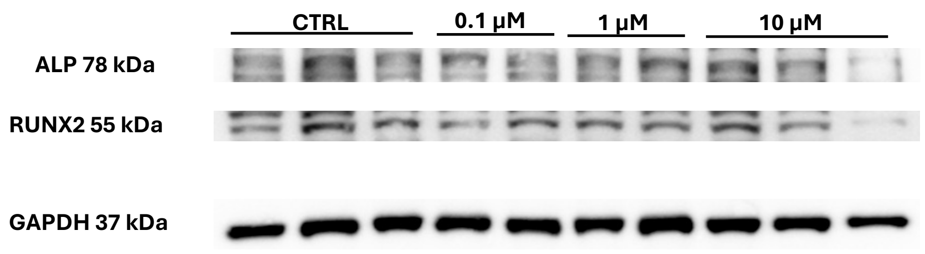

Supplement: Supplementary file 2 — Uncropped Western Blots [file 41420_2025_2863_MOESM2_ESM.pdf]
